# Phenotypically silent Cre recombination within the postnatal ventricular conduction system

**DOI:** 10.1371/journal.pone.0174517

**Published:** 2017-03-30

**Authors:** Samadrita Bhattacharyya, Minoti Bhakta, Nikhil Vilas Munshi

**Affiliations:** 1 Department of Internal Medicine (Cardiology Division), UT Southwestern Medical Center, Dallas, TX, United States of America; 2 Department of Molecular Biology, UT Southwestern Medical Center, Dallas, TX, United States of America; 3 McDermott Center for Human Growth and Development, UT Southwestern Medical Center, Dallas, TX, United States of America; 4 Center for Regenerative Science and Medicine, UT Southwestern Medical Center, Dallas, TX, United States of America; Baylor College of Medicine, UNITED STATES

## Abstract

The cardiac conduction system (CCS) is composed of specialized cardiomyocytes that initiate and maintain cardiac rhythm. Any perturbation to the normal sequence of electrical events within the heart can result in cardiac arrhythmias. To understand how cardiac rhythm is established at the molecular level, several genetically modified mouse lines expressing Cre recombinase within specific CCS compartments have been created. In general, Cre driver lines have been generated either by homologous recombination of Cre into an endogenous locus or Cre expression driven by a randomly inserted transgene. However, haploinsufficiency of the endogenous gene compromises the former approach, while position effects negatively impact the latter. To address these limitations, we generated a Cre driver line for the ventricular conduction system (VCS) that preserves endogenous gene expression by targeting the Contactin2 (Cntn2) 3’ untranslated region (3’UTR). Here we show that *Cntn2*^*3’UTR-IRES-Cre-EGFP/+*^ mice recombine floxed alleles within the VCS and that Cre expression faithfully recapitulates the spatial distribution of Cntn2 within the heart. We further demonstrate that Cre expression initiates after birth with preservation of native Cntn2 protein. Finally, we show that *Cntn2*^*3’UTR-IRES-Cre-EGFP/+*^ mice maintain normal cardiac mechanical and electrical function. Taken together, our results establish a novel VCS-specific Cre driver line without the adverse consequences of haploinsufficiency or position effects. We expect that our new mouse line will add to the accumulating toolkit of CCS-specific mouse reagents and aid characterization of the cell-autonomous molecular circuitry that drives VCS maintenance and function.

## Introduction

The Cardiac Conduction System (CCS) is composed of a set of specialized cardiomyocytes (CMs) that generate and propagate the electrical impulse required for contraction of the cardiac chambers [1–4]. The CCS is made of different constituents that perform particular roles. Pacemaker CMs in the Sino-Atrial Node (SAN), located at the junction of the right atrium (RA) and the superior vena cava, generate the electrical impulse, which is then rapidly conducted to the Atrio-Ventricular Node (AVN), where it undergoes a required delay. This delay in impulse conduction ensures proper atrial contraction and ventricular filling prior to ventricular contraction. The sole electrical bridge between the atrial and ventricular myocardium is composed of the fast-conducting Atrio-Ventricular Bundle (AVB), also referred to as the His bundle, which is connected to the AVN and continues through the crest of the ventricular septum. It propagates the electrical impulse to the right and left Bundle Branches (BB) and terminates at the right and left Purkinje Fiber (PF) network, which activates the ventricular myocardium. Collectively, these fast-conducting structures (AVB/His bundle, the right and left BB and the PF network) are called the Ventricular Conduction System (VCS). Any deviation from the normal electrical conduction can lead to abnormal heart rhythm [5]. Thus, maintaining normal cardiac rhythm becomes indispensable in the setting of structural heart disease, congenital heart defects, and inherited channelopathies. A particular example highlighting the importance of VCS components is the role of PF in triggering malignant ventricular arrhythmias [6–9].

The recent emergence of tissue-specific transgenic mouse models has vastly improved our understanding of the developing CCS, which can aid in developing targeted therapeutic strategies to treat arrhythmias. In particular, transgenic mice that express constitutive or inducible Cre recombinase within the CCS enable precise genetic manipulation *in vivo*. The first CCS-specific Cre driver mouse to be described was the Hcn4 CreERT2 knock-in line [10], but targeted insertion results in Hcn4 haploinsufficiency. Using this line, it was shown that Hcn4 not only labels the CCS but also serves as a dynamic marker of the first heart field [11–12]. A further caveat of the Hcn4 CreERT2 knock-in line is that recombinase activity also labels specific subsets of endothelium at distinct developmental stages. To circumvent the issue of haploinsufficiency, an independent group described the generation of a tamoxifen-inducible Hcn4-CreERT2 (BAC) transgenic mouse [13]. Perhaps due to Cre expression driven by a transgene rather than endogenous regulatory elements, however, tamoxifen-induced Cre activity is detected within the entire CCS after birth [13]. To contend with Hcn4 expression outside of pacemaker tissues, another group constructed a constitutive Shox2 Knock-In (KI) Cre allele to manipulate genes in the developing SAN [14]. This Cre line demonstrates specificity for pacemaker tissues, but insertion of Cre into the Shox2 locus also results in haploinsufficiency. Nevertheless, the availability of several Cre lines from which to choose provides investigators with multiple options for manipulating CCS genes *in vivo*.

In order to explore the VCS, several additional Cre driver lines have recently been developed. For example, inducible Cx40-CreERT2 mice, which carry a CreERT2-IRESmRFP cassette in the Connexin 40 (Cx40) locus, display reporter expression in the VCS and arterial endothelial cells upon tamoxifen induction [15]. Given that the Cx40-CreERT2 allele disrupts endogenous coding exons, another group created a minK:CreERT2 bacterial artificial chromosome (BAC) transgenic mouse line that specifically labels the AVN, AVB, and BB of transgenic mice following tamoxifen administration [16]. Thus, there exist at least two Cre lines capable of VCS-specific recombination, but issues of haploinsufficiency secondary to recombination and ectopic Cre expression due to transgene position effects remain to be addressed.

The Contactin *2* (Cntn2) gene encodes for a GPI-anchored neuronal membrane protein [17] that was recently identified as a specific marker of the VCS [18]. Early studies showed an important role for Contactin 2 in axon extension and guidance, fasciculation, and myelination during normal neurogenesis [19–21]. A later report investigated the contribution of Contactin 2 to locomotor recovery and successful regrowth of axons after spinal cord injury in adult zebrafish [22]. In the study by Pallante et al. [18], global gene expression patterns in PF-enriched and PF-depleted samples from CCS-lacZ hearts were compared by microarray analysis. The Cntn2 transcript showed the highest level of enrichment, and Cntn2 protein was localized to the sarcomlemma of murine CCS cells and isolated canine PF cells. Thus, Contactin2 represents an ideal target gene for creating genetically modified mice to manipulate the VCS. Aside from Cntn2, a recent study identified the transcription factor Etv1 to be highly abundant in pectinated atrial myocardium (PAM) and VCS myocytes, and *Etv1*^*nlz/+*^ KI reporter mice specifically label PAM and VCS myocytes within the heart [23]. Despite the existence of these above-mentioned transgenic lines, the precise cellular and molecular mechanisms controlling VCS function remain incompletely understood due in part to haploinsufficiency and inadequate specificity of the available Cre recombinase lines.

In this study, we describe a novel KI-Cre reporter mouse strain generated by targeted recombination of the *Cntn2* gene that precisely labels the VCS. To circumvent the various limitations of existing VCS Cre lines, we genetically engineered a new constitutive KI-Cre mouse model that enables specific manipulation of the VCS without any obvious phenotypic consequences. An *IRES-Cre-EGFP* KI cassette was incorporated in the 3’UTR of the *Cntn2* locus such that there is unperturbed bi-cistronic expression of Cntn2 protein and a Cre-EGFP fusion protein under the control of the endogenous regulatory elements. We show that *Cntn2*^*3’UTR-IRES-Cre-EGFP/+*^ mice recombine floxed alleles within the VCS and that Cre expression faithfully recapitulates the spatial distribution of Cntn2 within the heart. By monitoring Cre-dependent fluorescent reporter expression, we found that the *Cntn2*^*3’UTR-IRES-Cre-EGFP/+*^ allele is activated in the heart only after birth. We also confirmed that *Cntn2*^*3’UTR-IRES-Cre-EGFP/+*^ mice do not have any cardiac structural or functional defects. We believe that *Cntn2*^*3’UTR-IRES-Cre-EGFP/+*^ mice will serve as an invaluable tool to investigate the complex cellular and molecular machinery that drives VCS development. In turn, we expect that such insight will eventually pave the way for designing targeted therapies for ventricular arrhythmias and other conduction anomalies.

## Material and methods

### Generation of Cntn2^3’UTR-IRES-Cre-EGFP/+^ Knock-In (KI) mice

**Fig 1:**
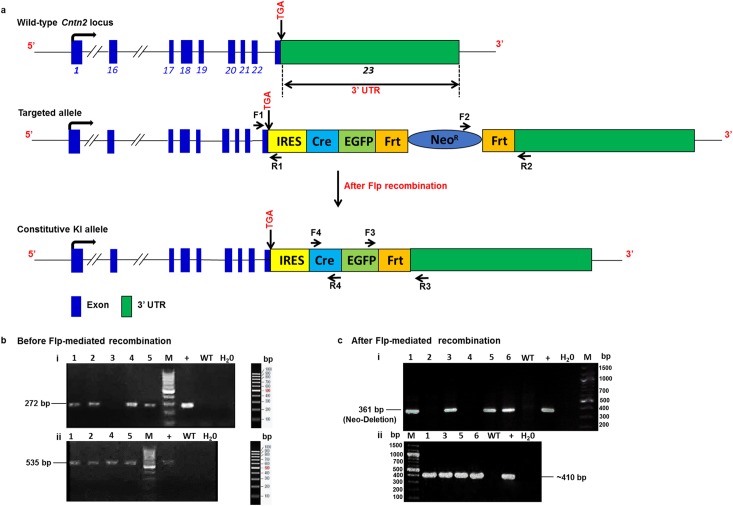
Genomic architecture of the *Cntn2*^*3’UTR-IRES-Cre-EGFP/+*^ allele. a) Schematic of the endogenous *Contactin-2 (Cntn2)* locus comprising 23 exons as indicated by blue numbered blocks. Green block denotes the 3’UTR of the *Cntn2* gene located in exon 23. The black right-handed arrow indicates the *Cntn2* transcriptional start site driven by endogenous regulatory elements. The *IRES-Cre-EGFP-FRT-neo-PGK-FRT* KI cassette was targeted to the 3’ UTR of the *Cntn2* locus to ensure unperturbed bi-cistronic expression of endogenous Cntn2 protein and a Cre-EGFP fusion protein under the control of the endogenous regulatory elements upon FLP recombination. b) PCR genotyping of mice before FLP-mediated recombination using the indicated primer sets to amplify the 5’ (i) and 3’ (ii) insertion sites. In this example, 4 out of 5 pups were positive by F1-R1 genotyping, and these 4 were subjected to the second round of genotyping using F2-R2 primer sets. C) PCR genotyping of mice after FLP-mediated recombination using the indicated primer sets to amplify across the deleted *Neo*^*R*^ cassette (i) and the *Cre* coding sequence (ii). In this example, 4 out of 6 pups were positive by F3-R3 genotyping, and these 4 were subjected to the second round of genotyping using F4-R4 primer sets.

We constructed a targeting vector containing an *IRES-Cre-EGFP-FRT-neo-PGK-FRT* KI cassette flanked by Homology Arms (HA) to ensure efficient Homologous Recombination (HR) within the 3’ UTR of the endogenous mouse *Cntn2* locus (details of the strategy shown in Fig 1A). The fidelity of the targeting construct was verified prior to introduction into Embryonic Stem (ES) cells by direct Sanger sequencing. Genetically modified mice were generated by standard methodology (Cyagen Biosiences Inc., Santa Clara, CA). Briefly, the targeting vector was electroporated into ES cells. The ES cell clones with correct HR were selected for by Neomycin (G418) resistance (*Neo*^*r*^) and screened by Polymerase Chain Reaction (PCR). ES cells were expanded, and injected blastocysts were implanted into pseudo-pregnant mice. In order to identify F1 mice with the recombined allele, standard PCR screening was used with F1-R1 and F2-R2 primer sets designed to identify the constitutive KI allele as shown in Fig 1A. The final PCR screening to verify *Neo*^*r*^ deletion was performed using primers F3-R3 designed as shown in Fig 1A. Following generation of genetically-modified mice, the 5’ and 3’ targeting construct insertion sites were re-verified by direct Sanger sequencing (S1 Fig). Sequences of all genotyping primers are provided in S1 Table.

### Commercial mouse strains

The FLP deleter strain (Strain number 009086) [24] and *R26R*^*tdTomato/tdTomato*^ reporter (Strain number 007914) [25] mice were obtained from the Jackson Laboratory (Bar Harbor, ME). For identification of the FLP allele and the *R26R* allele, we used two distinct primer sets (labelled Flp and R26R) which are enlisted in S1 Table. The newly generated KI reporter strain was maintained in a *C57BL/6* background. All experimental procedures with animals were approved by the Institutional Animal Care and Use Committee at UT Southwestern Medical Center. All mice were euthanized using methods in accordance with the Panel on Euthanasia of the American Veterinary Medical Association. Briefly, mice were euthanized by CO2 inhalation, and a secondary method, such as cervical dislocation or decapitation, was used to confirm death in the animal.

### Cloning

In order to verify appropriate genomic targeting and the fidelity of the recombined junctions, a high-fidelity thermostable DNA polymerase (Phusion; Thermo Scientific, #F-530S) was used to amplify the 5’ and 3’ KI cassette boundaries with primer sets F1-R1 and F3-R3, respectively. PCR fragments were inserted into the pCR-2.1 cloning vector and directly Sanger sequenced.

### Epifluorescent microscopy

Whole mount fluorescent images of whole embryos and organs were acquired with Zeiss Stemi SV11 dissection microscope equipped with epifluorescent and bright field illuminators and Optronics Macrofire camera setup. Objectives used were 0.63X and 1.6X, and scale bars were drawn based on the objective and zoom factor used.

### Immunostaining

Freshly dissected mouse hearts were fixed in 4% PFA at 4°C, then equilibrated in 10% sucrose, followed by 20% sucrose overnight at 4°C and embedded in Tissue Freezing Medium. Cryosections, 8μM thick, were acquired and then permeabilized for 20 minutes in 0.3% Triton X-100. Next, the sections were blocked for 10 minutes at 25°C in 1X Universal Blocking Reagent (Biogenex Laboratories). Slides were then incubated overnight at 4°C with the primary antibody followed by incubation for 1 hour at 25°C in the dark with the appropriate combination of secondary antibody and mounted with Vectashield with the nuclear counter stain DAPI (VectorLabs). All steps were performed in a dark humidified chamber.

### Antibodies

The following primary antibodies were used [species, target, dilution, company, and/or product number]: rabbit anti-Cx40, 1:250 (Alpha Diagnostic International); goat anti-Cntn2, 1:100 for immunofluorescence (IF) staining and 1:1000 for WB analysis (R&D Systems); mouse mAb α-Tubulin (DM1A), 1:1000 for WB analysis (Cell-Signaling Technology, #3873); rabbit polyclonal anti-Hcn4, 1:200 for IF staining and WB analysis (Alomone Labs, #APC-052); mouse anti-sarcomeric actinin, 1:200 (Sigma); and Rabbit anti-Myl2 Polyclonal antibody, 1:100 (ProteinTech). The following fluorophore-conjugated secondary antibodies were used: Alexa Fluor 488 Goat Anti-Rabbit IgG (H+L) Antibody, 1:400 (Invitrogen); Alexa Fluor 488 Rabbit Anti-Mouse IgG (H+L) Antibody, 1:400 (Invitrogen); Alexa Fluor 488 Rabbit Anti-Goat IgG (H+L) Antibody, 1:400 (Abcam). Antibodies were routinely diluted in 1X Universal Blocking Reagent at the appropriate concentration.

### Western blot analysis

For tissue samples, whole hearts were collected from P42 *Cntn2*^*3’UTR-IRES-Cre-EGFP/+*^ and WT mice (n = 3 for each group) and immediately snap frozen in liquid nitrogen. Pooled heart samples were then pulverized into a fine powder and homogenized in pre-chilled RIPA buffer (50 mM Tris pH 7.5, 150 mM NaCl, 1% NP-40, 0.5% sodium deoxycholate, 0.1% sodium dodecyl sulfate) supplemented with protease and phosphatase inhibitors. Further tissue disruption was carried out by three freeze-thaw cycles for 10 minutes each. The tissue lysate was clarified by centrifugation at 1000xg for 5 minutes prior to use. Protein-normalized samples were run on a 6% denaturing polyacrylamide gel and transferred to a PVDF membrane (Bio-Rad) overnight at 4°C. The membrane was blocked with 5% BSA in PBS with 0.1% Tween-20 prior to incubation with primary and secondary antibodies. Blots were developed with a chemiluminescent detection system (Santa Cruz), and individual bands were quantified using ImageJ software.

### Acetylcholinesterase staining

Heart cryosections (8mm thick) were fixed in 0.2% glutaraldehyde for 10 minutes at 25°C. The sections were washed thoroughly in sterile water before incubation with staining solution at 37°C overnight. Staining solution consists of 0.5mg/ml Acetylthiocholine iodide (#A5715, Sigma), 0.06N sodium acetate (#110191, Sigma), 0.1N acetic acid, 0.1M sodium citrate (#SX0445-1, VWR), 30mM cupric sulfate (#209198, Sigma), 4mM iso-OMPA (#T1505, Sigma), and 0.5M potassium ferricyanide. The slides were rinsed in sterile water the following day, counterstained in eosin, dehydrated and cleared in EtOH followed by Xylene. The slides were mounted in Permount, and images were captured using a Leica DM2000 upright compound microscope with Optronics Microfire camera with 10X and 20X objectives.

### Confocal microscopy

Confocal images of immunostained heart sections were acquired using Nikon A1R+ scanning confocal system. Objectives used were 10X and 20X (dry). Excitation laser filters used were 488 nm line; green fluorescence and 555 nM line; red fluorescence. Images were analyzed using NIS Elements Viewer v4.2 software, ImageJ, and Adobe Photoshop CS6 Extended software.

### Echocardiography

Vevo2100 high-resolution digital imaging platform was used to evaluate the mechanical properties of P42 hearts from both groups of animals by M-mode echocardiography in awake animals. VevoStrain™ Analysis software was used to process the data and calculate heart function parameters, including Heart Rate, Fractional Shortening, Ejection Fraction, and Cardiac Output.

### Electrocardiography (ECG)

Surface electrocardiography was performed on mice at the indicated ages under mild anesthesia with 2% isoflurane in 200mL/min oxygen. The subcutaneous leads were placed in the conventional lead II position. ECG was recorded by BioAmp connected to the Powerlab, and data were processed with Chart5 (ADInstruments, CO, U.S.A). For pharmacological stress experiments, intraperitoneal injection of isoproterenol was given to individual mice during ECG recordings, and traces were obtained before and after isoproterenol adminstration.

### Histology

Freshly dissected hearts were fixed overnight in 10% Neutral Buffered Formalin. Routine Hematoxylin and Eosin (H&E) staining was performed on paraffin-embedded heart sections. Images were captured using Leica DM2000 upright compound microscope with Optronics Microfire camera. Objective used was 1.25X.

### Statistical analysis

Data are presented as Mean ± S.E.M. Statistical analysis between groups of animals was performed using an unpaired Mann-Whitney non-parametric test, and probability values (p-value) >0.05 were not considered statistically significant. The statistical calculations were performed using GraphPad Prism 6 software.

## Results

### Cntn2^3’UTR-IRES-Cre-EGFP/+^ mice recombine loxP sites within the VCS

The *Cntn2* gene (OMIM ID: 190197) contains 23 exons spanning approximately 40 kilobases (kb) with the ATG start codon in exon 2 and the TGA stop codon in exon 23 [26]. The mCntn2 gene (GenBank accession number NM_177129.5; Ensembl ID ENSMUSG00000053024) is located on mouse chromosome 1. In order to generate a highly specific and sensitive mouse model to facilitate studies of the VCS, the *Cntn2* locus was targeted for Cre insertion. The targeting vector was designed to have 5’ and 3’ Homology Arms (HA) with an *IRES-Cre-EGFP-FRT-neo-PGK-FRT* KI cassette to ensure proper HR into the 3’ UTR of the *Cntn2* locus. This targeting strategy ensures unperturbed bi-cistronic expression of endogenous Cntn2 protein and a Cre-EGFP fusion protein under the control of the endogenous regulatory elements upon FLP recombination (Fig 1A). We used standard Polymerase Chain Reaction (PCR) screening to verify the following: 1) 5’ recombination using primer set F1-R1 designed to amplify a 272 bp fragment from the *Cntn2* locus and the 5’ end of the KI cassette, 2) 3’ recombination using primer set F2-R2 designed to amplify a 535 bp product from the *Cntn2* locus and the 3’ end of the KI cassette, 3) *neo-PGK* cassette removal with primer sets F3-R3 designed such that a band of 361bp is detected only upon *neo* deletion by FLP-mediated recombination, and 4) *Cre* sequence with primer sets F4-R4 that amplify a 408bp fragment within the coding sequence (Fig 1B and 1C). Furthermore, appropriate genomic targeting and fidelity of the recombined 5’ and 3’ junctions were confirmed by high-fidelity PCR coupled with direct Sanger sequencing of the KI cassette boundaries as shown in S1 Fig.

**Fig 2:**
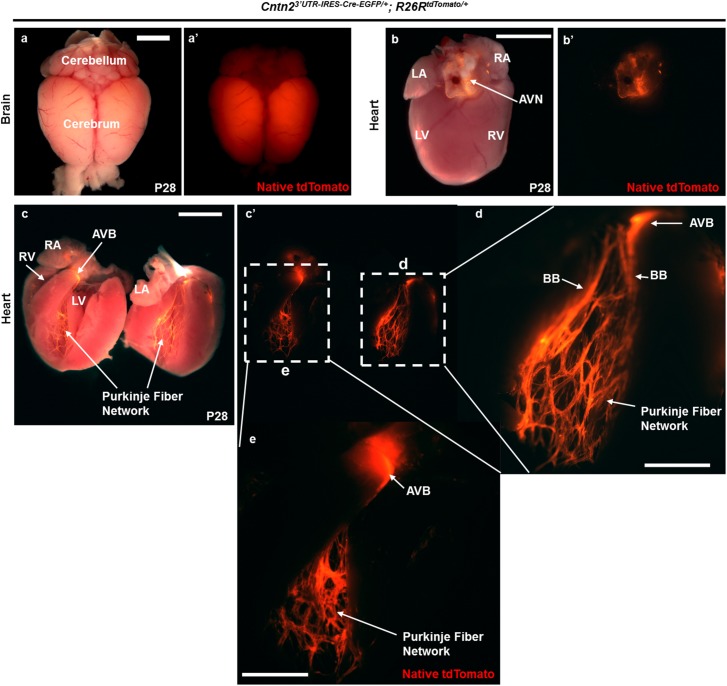
*Cntn2*^*3’UTR-IRES-Cre-EGFP/+*^*; R26R*^*tdTomato/+*^ mice display robust reporter expression in the VCS. (a-e) *Cntn2*^*3’UTR-IRES-Cre-EGFP/+*^ mice were bred with *R26R*^*tdTomato/tdTomato*^ reporter mice to characterize the *Cntn2*^*3’UTR-IRES-Cre-EGFP/+*^ allele by monitoring tdTomato expression at P28 by whole mount fluorescence imaging (a and a’) in brain (dorsal view, used as a positive control) where endogenous Contactin 2 is known to be expressed abundantly and (b-e) in heart. We monitored tdTomato expression in at least 15 independent *Cntn2*^*3’UTR-IRES-Cre-EGFP/+*^*; R26R*^*tdTomato/+*^ mice from multiple litters in comparison to 12 WT control littermates. (b and b’) Native tdTomato fluorescence was visualized at the Atrio-Ventricular Junction (AVJ) by whole-mount microscopy following Cre recombination of the *R26R* locus. (c-e) A P28 mouse heart was sectioned grossly in the four-chamber orientation to visualize native tdTomato expression in the VCS. (c and c’) Robust tdTomato fluorescence was observed in the Atrio-Ventricular Bundle (AVB), right and left Bundle Branches (BB), and right and left Purkinje Fiber (PF) network. Faint speckled fluorescent signal was also observed in the Right Atrium (RA) (not visible in the image). (a-c) Scale bar: 500 μM. (d and e) Higher magnification images of (d) right inlet in (c’) and (e) left inlet in (c’) to visualize the intricate structures of AVB and highly branched PF network in the adult mouse heart. Scale bar: 250 μm. LA, Left Atrium; LV, Left Ventricle; RV, Right Ventricle.

To characterize the expression pattern of the KI-*Cre* allele, *Cntn2*^*3’UTR-IRES-Cre-EGFP/+*^ mice were bred with the *R26R*^*tdTomato/tdTomato*^ reporter strain. Cre dependent tdTomato expression was monitored at different stages of murine development. Consistent with Cntn2 expression in the central nervous system [27, 28], we observed robust fluorescent reporter expression in the brain at P28 (Fig 2A). In addition, we noted native tdTomato fluorescence in the heart at P28. Using epifluorescence imaging on freshly isolated P28 *Cntn2*^*3’UTR-IRES-Cre-EGFP/+*^*; R26R*^*tdTomato/+*^ hearts (n = 15), we visualized reporter expression on the dorsal surface of the heart at the Atrio-Ventricular Junction (AVJ) upon Cre-mediated recombination of the *R26R* locus (Fig 2B). The same heart was further sectioned grossly to permit detailed visualization of tdTomato in specific regions and structures within the heart. The cells of the VCS exhibited bright and specific fluorescent labelling (Fig 2C and 2C’). Higher magnification imaging of the four chambers of an adult *Cntn2*^*3’UTR-IRES-Cre-EGFP/+*^*; R26R*^*tdTomato/+*^ heart allowed appreciation of even finer and more complex structures of the AVB, right and left BB, and PF network (Fig 2D and 2E). To ensure that reporter expression was Cre-dependent, we sectioned multiple adult *Cntn2*^*+/+*^*; R26R*^*tdTomato/+*^ (hereafter referred to as wild-type [WT]) animals and found no evidence of tdTomato expression (see S4, S6C and S6D and S7 Figs). The litter size and sex-distribution also followed the expected Mendelian ratio (data not shown), suggesting that *Cntn2* KI-allele does not have any major phenotypic consequences. Overall, we established a highly specific KI reporter mouse that labels key constituents of the VCS, namely the AVB-BB-PF system.

### Cntn2^3’UTR-IRES-Cre-EGFP/+^ mice specifically label VCS components

**Fig 3:**
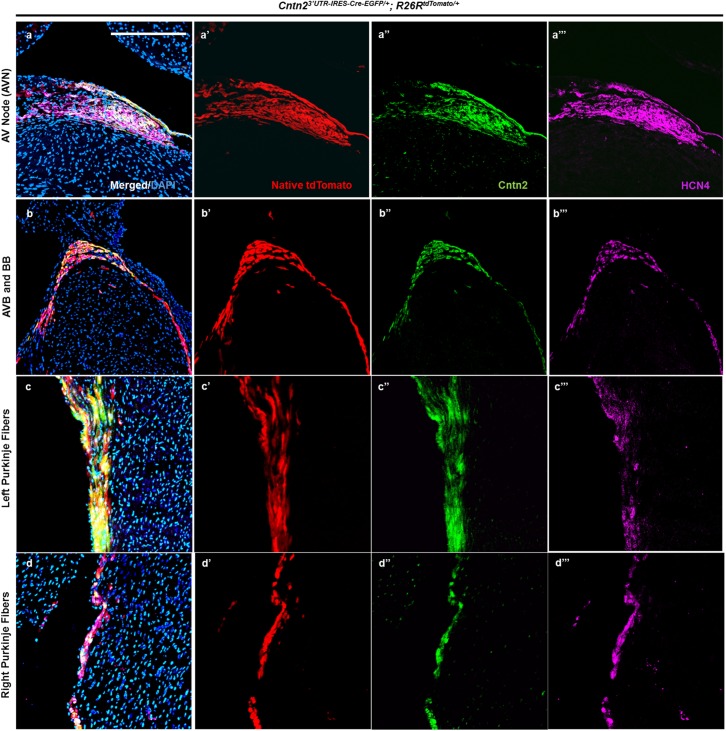
tdTomato co-localizes with VCS markers in *Cntn2*^*3’UTR-IRES-Cre-EGFP/+*^*; R26R*^*tdTomato/+*^ mice. (a-d) *Cntn2*^*3’UTR-IRES-Cre-EGFP/+*^ mice were crossed with *R26R*^*tdTomato/tdTomato*^ reporter mice, and P42 heart cryosections were imaged by confocal microscopy. (a, a’, a”, a”‘) High resolution confocal images of the AVN region demonstrates co-localization of tdTomato (a’, red), endogenous Cntn2 (a”, green), and endogenous Hcn4 (a”‘, magenta) with the merged image shown in (a). (b, b’, b”, b”‘) High specificity and complete overlap (b: merged signal) of native tdTomato (b’, red), endogenous Cntn2 (b”, green), and endogenous Hcn4 (b’”, magenta) expression upon Cre recombination in the AVB and BB. (c, c’, c”, c”‘) Confocal images of mouse heart sections showing that Cre recombined tdTomato cells of left PFs (c’, red) co-localize with endogenous Cntn2 (c”, green) and Hcn4 (c”‘, magenta). (d, d’, d”, d”‘) Images of serial *Cntn2*^*3’UTR-IRES-Cre-EGFP/+*^*; R26R*^*tdTomato/+*^ mice heart sections verified co-expression of endogenous Cntn2 (d”, green) and Hcn4 (d”‘, magenta) in recombined tdTomato cells (d’, red) of right PFs. Blue signal indicates nuclear counterstaining with DAPI. The cardiac anatomical location for each confocal micrograph is shown in S2A Fig. Scale bar: 100 μm.

To verify that *Cntn2*^*3’UTR-IRES-Cre-EGFP/+*^ mice label VCS structures, we stained P42 heart cryosections from *Cntn2*^*3’UTR-IRES-Cre-EGFP/+*^*; R26R*^*tdTomato/+*^ mice with different antibodies and chemical stains (details provided in Materials and Methods). First, we used Acetylcholinesterase (AChase) staining to anatomically map individual components of the CCS in *Cntn2*^*3’UTR-IRES-Cre-EGFP/+*^*; R26R*^*tdTomato/+*^ mice (S3C–S3F Fig). As expected, we observed labeling of distinct CCS components, including the AVN, AVB, BB, and PFs. Based on this anatomical map, we then performed immunostaining with antibodies for native Cntn2 and Hcn4, which marks all components of the CCS from late fetal to early adult stages [12]. By confocal microscopy, we observed precise and complete co-localization of endogenous Contactin 2, Hcn4, and tdTomato following Cre recombination in the AVN, AVB, BB, and PFs (Fig 3), suggesting that the *Cntn2*^*3’UTR-IRES-Cre-EGFP/+*^ allele faithfully marks the VCS. We also observed overlapping expression of tdTomato and Cx40 (S3A and S3B Fig), a well-established VCS marker, to provide additional support for the VCS specificity of the *Cntn2*^*3’UTR-IRES-Cre-EGFP/+*^ allele. Based on the anatomical location of tdTomato, co-localization with AChase staining, and co-expression with native Cntn2, Hcn4, and Cx40, we conclude that the *Cntn2*^*3’UTR-IRES-Cre-EGFP/+*^ allele faithfully marks the VCS.

In order to ensure structural integrity of the heart in *Cntn2*^*3’UTR-IRES-Cre-EGFP/+*^ animals, we performed Hematoxylin and Eosin (H&E) staining on P42 mice and did not visualize any obvious morphological defects (S2 Fig). Next, we wished to exclude the possibility that the *Cntn2*^*3’UTR-IRES-Cre-EGFP/+*^ allele perturbs the pattern of CCS marker expression. Therefore, we defined the anatomical layout of the CCS in WT mice by AChase staining and performed immunostaining for Hcn4, Cntn2, and Cx40 (S4 Fig). By comparing sections from *Cntn2*^*3’UTR-IRES-Cre-EGFP/+*^*; R26R*^*tdTomato/+*^ and WT mice, we found no obvious differences in the expression pattern of Hcn4, Cntn2, or Cx40 in various segments of the VCS (Fig 3, S3 and S4 Figs). Although these results strongly suggest that the *Cntn2*^*3’UTR-IRES-Cre-EGFP/+*^ allele does not perturb CCS marker expression, they are not quantitative. Therefore, we performed quantitative Western Blot analysis to confirm that endogenous Cntn2 and Hcn4 levels remain unchanged (S5 Fig). Furthermore, to establish that the allele does not affect more widely expressed cardiac markers, we stained sections from *Cntn2*^*3’UTR-IRES-Cre-EGFP/+*^*; R26R*^*tdTomato/+*^ and WT mice for the pan-cardiac α-actinin and the pan-ventricular Myl2 markers (S6 Fig). Importantly, these studies showed no alteration in α-actinin or Myl2 expression and confirmed that tdTomato^+^ cells are indeed cardiomyocytes of ventricular origin. Thus, our results demonstrate that the *Cntn2*^*3’UTR-IRES-Cre-EGFP/+*^ allele does not affect cardiac morphology, CCS structure, or ventricular myocyte marker expression. Collectively, we conclude that Cre-EGFP co-localizes with cellular markers of the VCS and that expression of important endogenous cardiac markers is preserved by targeted recombination of the *Cntn2* 3’UTR.

### The Cntn2^3’UTR-IRES-Cre-EGFP/+^ allele is activated in the heart after birth

**Fig 4:**
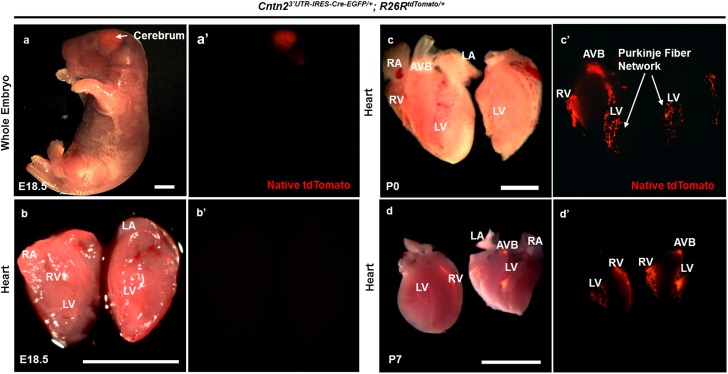
Cre-mediated recombination in *Cntn2*^*3’UTR-IRES-Cre-EGFP/+*^*; R26R*^*tdTomato/+*^ mice is only evident after birth. (a, a’) Whole mount fluorescent image of an E18.5 double heterozygous embryo with robust expression of the recombined reporter protein in the cerebrum. tdTomato expression in the brain was also seen at E16.5 (data not shown). (b, b’) Following microdissection of the heart at E18.5, native tdTomato signal was not observed. (c, c’) Double heterozygous P0 mouse heart after dissection revealed bright reporter protein expression in the AVB, right and left BB, and right and left PF network. P0 was the earliest point at which reporter expression was observed in the heart. (d,d’) Micro-dissected P7 double heterozygous hearts express tdTomato protein in the identical anatomical regions as described in (c,c’). The cardiac PF network appears fully developed by P7 based on fluorescent reporter expression. At least 8 hearts were dissected per group of animals at each time point. Scale bar: 500 μm.

To identify the developmental time point at which our newly generated *Cre* allele is activated in the murine heart, we assessed reporter expression in an E18.5 double heterozygous embryo. Although we observed robust expression of the recombined reporter protein in the cerebrum (Fig 4A and 4A’), native tdTomato signal was not seen following microdissection of the heart at E18.5 (Fig 4B and 4B’). In contrast, we noticed bright tdTomato fluorescence in the AVB, right and left BB, and right and left PF network at P0 (Fig 4C and 4C’). We also monitored tdTomato protein at P7 (Fig 4D and 4D’) and found that the fluorescent signal was localized to the identical anatomical regions described in Fig 4C and 4C’. For each developmental time point, we dissected at least 8 hearts in each group. From these data, we conclude that the *Cntn2*^*3’UTR-IRES-Cre-EGFP/+*^ allele mediates Cre-dependent recombination after birth and that the cardiac PF network appears fully developed by P7 based on tdTomato reporter expression.

We also tracked fluorescent labeling at earlier embryonic time-points (e.g. E16.5 and E17.5), but we found no tdTomato expression in the heart even after microdissection. However, robust tdTomato protein expression appeared in the brain at E16.5 and E17.5 (data not shown). Taken together, these results suggest that Cre is expressed in the brain of *Cntn2*^*3’UTR-IRES-Cre-EGFP/+*^ mice during embryogenesis, but cardiac expression initiates abruptly after birth. Furthermore, cellular labelling was entirely Cre dependent since we observed no tdTomato fluorescence in WT animals (n = 8 for each developmental time point) at E18.5, P0, P7 (S7 Fig), or P28 (data not shown). Overall, we found that P0 was the earliest point at which reporter expression was observed in the heart, suggesting that Cre activity in double heterozygous mice is evident only after birth.

### Cardiac function is maintained in Cntn2^3’UTR-IRES-Cre-EGFP/+^ mice

**Fig 5:**
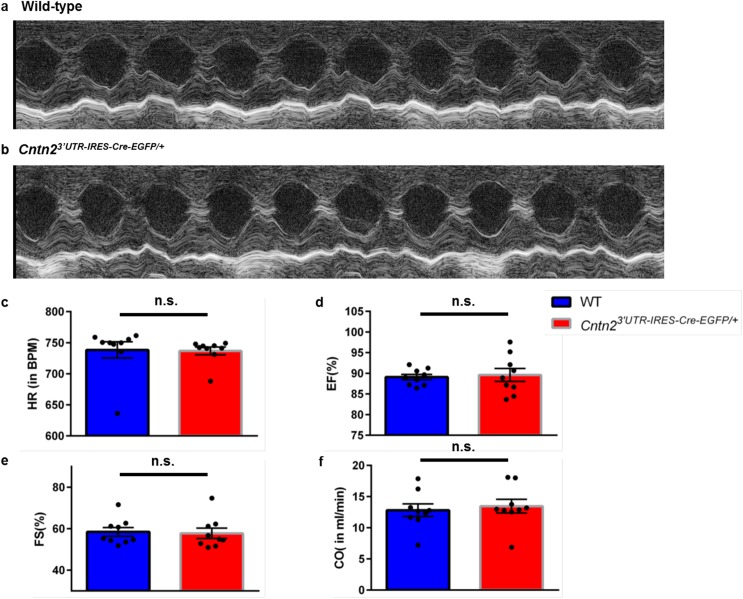
Cardiac mechanical function is preserved in *Cntn2*^*3’UTR-IRES-Cre-EGFP/+*^ mice. (a-b) M-mode echocardiography was performed in conscious mice to analyze heart function parameters in P42 (a) wild-type (WT) and (b) *Cntn2*^*3’UTR-IRES-Cre-EGFP/+*^ mice. Representative traces show normal heart function in both groups. (c) Heart Rate (HR) in Beats Per Minute (BPM), (d) Ejection Fraction (EF) as a percentage, (e) Fractional Shortening (FS) as a percentage, and (f) Cardiac Output (CO) in milliliters per minute were calculated for n = 9 animals in both groups. Black circles represent individual WT and *Cntn2*^*3’UTR-IRES-Cre-EGFP/+*^ mice used in this study. Blue and red bars represent mean values of each parameter in WT and *Cntn2*^*3’UTR-IRES-Cre-EGFP/+*^ mice, respectively. No statistically significant differences were observed between groups. ns, not significant.

A major theoretical advantage of our Cntn2 allele is that Cre-EGFP is driven by endogenous regulatory elements without affecting Cntn2 expression. To confirm that *Cntn2*^*3’UTR-IRES-Cre-EGFP/+*^ mice do not have any cardiac defects, we conducted a series of functional studies. In order to evaluate cardiac mechanical properties in *Cntn2*^*3’UTR-IRES-Cre-EGFP/+*^ mice, we performed M-mode echocardiography on conscious P42 WT and *Cntn2*^*3’UTR-IRES-Cre-EGFP/+*^ mice (n = 9 per group). We measured Heart Rate (HR), Ejection Fraction (EF), Fractional Shortening (FS), and Cardiac Output (CO) in both groups of animals (Fig 5A–5F). No statistically significant differences were observed between groups. Other heart function parameters like Stroke Volume, Left Ventricular Mass, Systolic and Diastolic Diameter and Volume were also calculated and appeared to be normal (data not shown).

**Fig 6:**
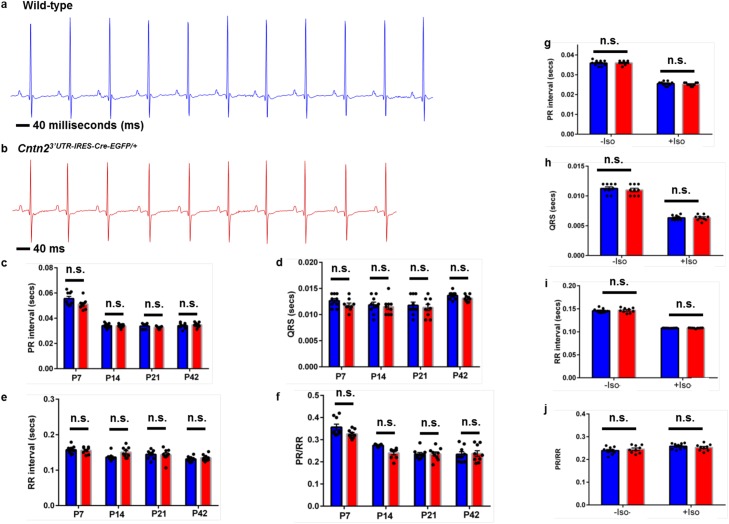
Cardiac electrical function is preserved in *Cntn2*^*3’UTR-IRES-Cre-EGFP/+*^ mice. (a-j) Serial surface lead II ECGs were recorded on both groups of mice (n = 9 per group) under mild anesthesia at P7, P14, P21, P28, and P42. Exemplary ECG traces for P42 (a) WT and (b) *Cntn2*^*3’UTR-IRES-Cre-EGFP/+*^ mice are shown. Scale size: 40 milliseconds (ms). (c) PR interval (in seconds), (d) QRS width (in seconds), (e) RR interval (in seconds), and (f) PR/RR ratio were measured at P7, P14, P21, and P42. At P28, (g) PR interval (in seconds), (h) QRS width (in seconds), (i) RR interval (in seconds), and (j) PR/RR ratio were measured pre- and post-isoproterenol (-/+ Iso) injection. Black circles represent individual WT and *Cntn2*^*3’UTR-IRES-Cre-EGFP/+*^ mice used in this study. Blue and red bars represent mean values for each parameter in WT and *Cntn2*^*3’UTR-IRES-Cre-EGFP/+*^ mice, respectively. No statistically significant differences were observed between groups. ns, not significant.

To determine if cardiac electrical parameters are similarly preserved in *Cntn2*^*3’UTR-IRES-Cre-EGFP/+*^ mice, we recorded serial surface lead II ECGs (details of the procedure in the Material and Methods section) on both groups of mice (n = 9 per group) under mild anesthesia at P7, P14, P21, P28, and P42. ECG traces appeared similar between WT and *Cntn2*^*3’UTR-IRES-Cre-EGFP/+*^ mice (Fig 6A and 6B). We measured PR interval, RR interval, QRS width, and PR/RR ratio and observed that electrical activity was maintained in both groups without any significant differences throughout the time course (Fig 6C–6F). Given that some cardiac phenotypes are unmasked during stress, we wished to exclude this possibility in our *Cntn2*^*3’UTR-IRES-Cre-EGFP/+*^ mice. Therefore, we subjected *Cntn2*^*3’UTR-IRES-Cre-EGFP/+*^ and WT mice (n = 9 per group) to pharmacological stress by intraperitoneal administration of isoproterenol, and ECGs were recorded before and after stimulation. We observed the expected PR shortening in both groups of mice (Fig 6G), demonstrating that injected isoproterenol functioned appropriately. Importantly, we observed no significant differences between *Cntn2*^*3’UTR-IRES-Cre-EGFP/+*^ and WT mice either before or after isoproterenol administration in any of the measured ECG parameters (Fig 6G–6J). Furthermore, we did not detect any non-sinus rhythms in either group before or after isoproterenol was given (see S8 Fig for representative ECG tracings). Collectively, these data demonstrate preserved cardiac mechanical and electrical function in *Cntn2*^*3’UTR-IRES-Cre-EGFP/+*^ mice.

## Discussion

Here we describe construction of a *Cntn2*^*3’UTR-IRES-Cre-EGFP/+*^ allele generated by targeted HR of the *Cntn2* locus. We successfully characterized this novel KI-*Cre* reporter mouse and demonstrated precise labelling of the VCS by co-expression of Cre-EGFP with established VCS markers. Importantly, we observed unperturbed expression of endogenous cardiac markers following targeted recombination of the *Cntn2* 3’UTR. By monitoring Cre-dependent fluorescent reporter expression, we found that the *Cntn2*^*3’UTR-IRES-Cre-EGFP/+*^ allele is activated in the heart only after birth. We also confirmed that *Cntn2*^*3’UTR-IRES-Cre-EGFP/+*^ mice do not have any cardiac structural or functional defects. Therefore, our mouse model will serve as a powerful *in vivo* tool for analyzing crucial factors governing VCS lineage specification and potentially identifying new therapeutic targets for ventricular arrhythmias and other fast cardiac conduction anomalies.

Despite the emergence of several CCS-specific mouse models [11,13–16], studying the cell-autonomous molecular circuitry that regulates VCS function has remained challenging due to specific disadvantages of currently available Cre driver lines. Specifically, many existing CCS Cre lines result in haploinsufficiency, cause non-specific expression, and/or suffer from position-dependent transgene expression that potentially impact the interpretation of conditional knockout studies. Thus, we expect that our *Cntn2*^*3’UTR-IRES-Cre-EGFP/+*^ mouse model will address many of these limitations and facilitate future studies of VCS specification, patterning, and activity. Additionally, the *Cntn2*^*3’UTR-IRES-Cre-EGFP/+*^ mouse line can serve as an important tool to investigate the molecular and cellular networks governing neurogenesis as we also show faithful recapitulation of reporter expression in the brain.

Although the complex molecular circuitry that regulates VCS function and maintenance remains incompletely understood, recent work has begun to shed light on this important topic [29, 30]. A seminal study identified a transcription factor (TF) cascade involving *Tbx5*, *Nkx2-5*, and *Id2* that governs developmental specification of ventricular myocytes towards a VCS fate [30]. Furthermore, recent work has identified additional TFs that regulate VCS function, such as Etv1 [23]. However, neither of these studies explored cell-autonomous TF function, since VCS-specific Cre lines were not used to conditionally delete the TF of interest. In this regard, Moskowitz and colleagues recently found that Tbx5 is required within the VCS for maintenance of normal cardiac rhythm using the mink(BAC):CreERT2 KI strain to conditionally delete Tbx5 [31]. We hope that our novel KI-*Cre* allele will add to this emerging body of literature by facilitating future mechanistic studies aimed at deciphering the cell autonomous function of TFs within the VCS.

## Supporting information

S1 Fig Proper targeting of the Cntn2 genomic locus and preserved fidelity of the recombined junctions. Representative Sanger sequencing results confirm appropriate targeting of the Cntn2 locus and the fidelity of the (a) 5’ and (b) 3’ KI cassette boundaries
(TIF)

S2 Fig Structural integrity is preserved in *Cntn2*^*3’UTR-IRES-Cre-EGFP/+*^ hearts. (a, b) Hematoxylin and Eosin (H&E) stained heart sections in a four-chamber orientation from (a) *Cntn2*^*3’UTR-IRES-Cre-EGFP/+*^ and (b) WT animals at P42 showing the approximate anatomical location (1.25 X objective, black dotted inlets) of the detailed cardiac structures (dashed boxes) represented in Figs 3A–3D, S3, S4 and S6. (a’, b’) Higher magnification (20X objective, blue dotted inlets) images of H&E stained sections shown in (a, b) for confirming structural and morphological integrity of (a) *Cntn2*^*3’UTR-IRES-Cre-EGFP/+*^ and (b) WT animals respectively. Scale bars: 500 μm.
(TIF)

S3 Fig Co-localization of tdTomato with additional VCS markers in *Cntn2*^*3’UTR-IRES-Cre-EGFP/+*^*; R26R*^*tdTomato/+*^ reporter mice. (a,b) High-power confocal images of consecutive heart sections of P42 *Cntn2*^*3’UTR-IRES-Cre-EGFP/+*^*; R26R*^*tdTomato/+*^ mice to corroborate the co-expression (a and b) of the landmark Purkinje cell marker Cx40 (a’ and b’ in green) and native tdTomato (a” and b”‘ in red) in the right BB (a) and left PF network (b). Blue signal indicates nuclear counterstain by DAPI. (c-f) Acetylcholinesterase (AChase) staining of sister sections from a P42 *Cntn2*^*3’UTR-IRES-Cre-EGFP/+*^*; R26R*^*tdTomato/+*^ heart, demonstrating clear identification of the AVN (c), right PFs (d), AVB and BBs (e), and left PFs (f). Scale bars: (a-b) 100 μm; (c-f) 500 μm. The precise anatomical location of the structures labeled in (a-f) are shown at lower magnification in S2A Fig.
(TIF)

S4 Fig VCS marker expression in WT mice. Littermates of *Cntn2*^*3’UTR-IRES-Cre-EGFP/+*^*; R26R*^*tdTomato/+*^ mice at P42 were used as WT controls. (a, a’, a”, a”‘) (a) Merged image of (a’) tdTomato (red), (a”) Cntn2 (green), and (a”‘) Hcn4 (magenta) in the AV Bundle (AVB). (b, b’, b”, b”‘) (b) Merged image of (b’) tdTomato (red), (b”) Cntn2 (green), and (b”‘) Hcn4 (magenta) in the right Bundle Branch (R-BB). (c, c’, c”, c”‘) (c) Merged image of (c’) tdTomato (red), (c”) Cntn2 (green), and (c”‘) Hcn4 (magenta) in the right Purkinje Fibers (R-PF). (d, d’, d”) (d) Merged image of (d’) tdTomato (red) and (d”) Cx40 (green) in the left Purkinje Fibers (L-PF). As expected, we observed no evidence of tdTomato expression in the VCS (a’-d’), indicating a lack of recombination in WT animals. (e-h) AChase staining of sister sections clearly demarcating the (e) AVB, (f) left and right BBs, (g) RBB, and (h) left PFs. (a-d) Blue signal indicates nuclear counterstaining by DAPI. Scale bars: (a-d)100μm; (e-h) 500μm. The precise anatomical location of the structures labeled in (a-f) are shown at lower magnification in S2B Fig.
(TIF)

S5 Fig Endogenous Cntn2 and Hcn4 protein expression is preserved in *Cntn2*^*3’UTR-IRES-Cre-EGFP/+*^ mice. (a-b) Total protein was extracted from whole heart tissue of P42 *Cntn2*^*3’UTR-IRES-Cre-EGFP/+*^ and WT mice (n = 3 hearts for each group), and the amount of total protein was normalized across samples with α-Tubulin serving as a loading control. a) Cntn2 protein expression was assessed by Western Blot analysis. a’) Quantification of the blots in (a) by ImageJ confirmed that Cntn2 expression is maintained in *Cntn2*^*3’UTR-IRES-Cre-EGFP/+*^ mice. b) Hcn4 protein expression was evaluated by Western Blot analysis. b’) Quantification of the blots in (b) verified that Hcn4 expression is prserved in *Cntn2*^*3’UTR-IRES-Cre-EGFP/+*^ mice. Quantification was based on two independent blots for each target protein. Blue and red bars represent protein quantification for WT and *Cntn2*^*3’UTR-IRES-Cre-EGFP/+*^ mice, respectively.
(TIF)

S6 Fig Cardiac marker expression is maintained in *Cntn2*^*3’UTR-IRES-Cre-EGFP/+*^*; R26R*^*tdTomato/+*^ mice. High-power confocal images of sections derived from *Cntn2*^*3’UTR-IRES-Cre-EGFP/+*^*; R26R*^*tdTomato/+*^ (a-b) and WT (c-d) mice at P42. Sections were stained for either the pan-ventricular marker Myl2 (a’ and c’) or the pan-cardiac marker α-actinin (b’ and d’). No obvious differences in either marker were found between the two groups of mice. As expected, tdTomato expression was only observed in the presence of the *Cntn2*^*3’UTR-IRES-Cre-EGFP/+*^ allele (compare a”-b” with c”-d”). Blue signal indicates nuclear counterstaining by DAPI. Scale bars: 100μm. The precise anatomical location of the structures labeled in (a-d) are shown at lower magnification in S2A and S2B Fig.
(TIF)

S7 Fig WT animals display no fluorescent reporter expression at different stages of murine development. (a, a’) Whole mount fluorescent imaging of an E18.5 WT embryo exhibit no reporter expression indicating no recombination. We also confirmed no tdTomato expression in the brain of an E16.5 WT embryo (data not shown). (b, b’) After microdissection of a WT heart at E18.5, no reporter signal was observed. (c, c’) WT P0 mice hearts after dissection revealed no reporter protein expression. (d, d’) Microdissected P7 WT control hearts do not express tdTomato protein in any cardiac structures. n = 8 hearts were dissected for each developmental time point. Scale bar: 500 μm.
(TIF)

S8 Fig *Cntn2*^*3’UTR-IRES-Cre-EGFP/+*^ mice show no evidence of non-sinus rhythms at baseline or after pharmacological stress. (a-b) Surface lead II ECGs were recorded continuously before and after pharmacological stress with isoproterenol. Representative ECG tracings are shown for (a) WT and (b) *Cntn2*^*3’UTR-IRES-Cre-EGFP/+*^ mice. No non-sinus rhythms were observed in either group before or after isoproterenol injection (n = 9 per group). Scale bar: 20 milliseconds (ms).
(TIF)

S1 Table List of genotyping primers. (DOCX)
