# Supplementary figures and images for: Phenotypically silent Cre recombination within the postnatal ventricular conduction system
Source: PLoS One. 2017 Mar 30;12(3):e0174517. doi: 10.1371/journal.pone.0174517 (PMC5373586; doi:10.1371/journal.pone.0174517)

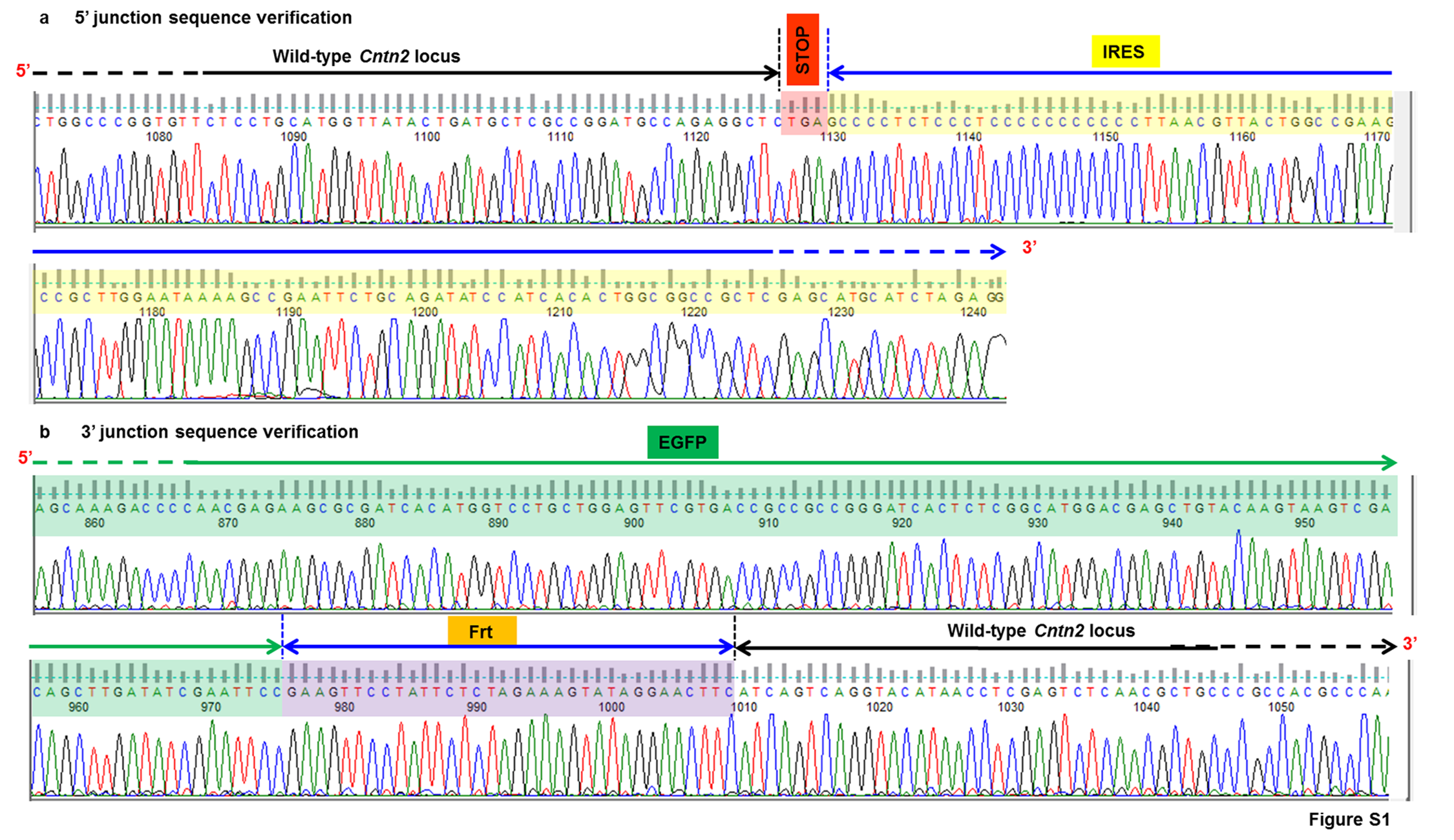

Supplement: S1 Fig — Representative Sanger sequencing results confirm appropriate targeting of the Cntn2 locus and the fidelity of the (a) 5’ and (b) 3’ KI cassette boundaries (TIF) [file pone.0174517.s001.tif]

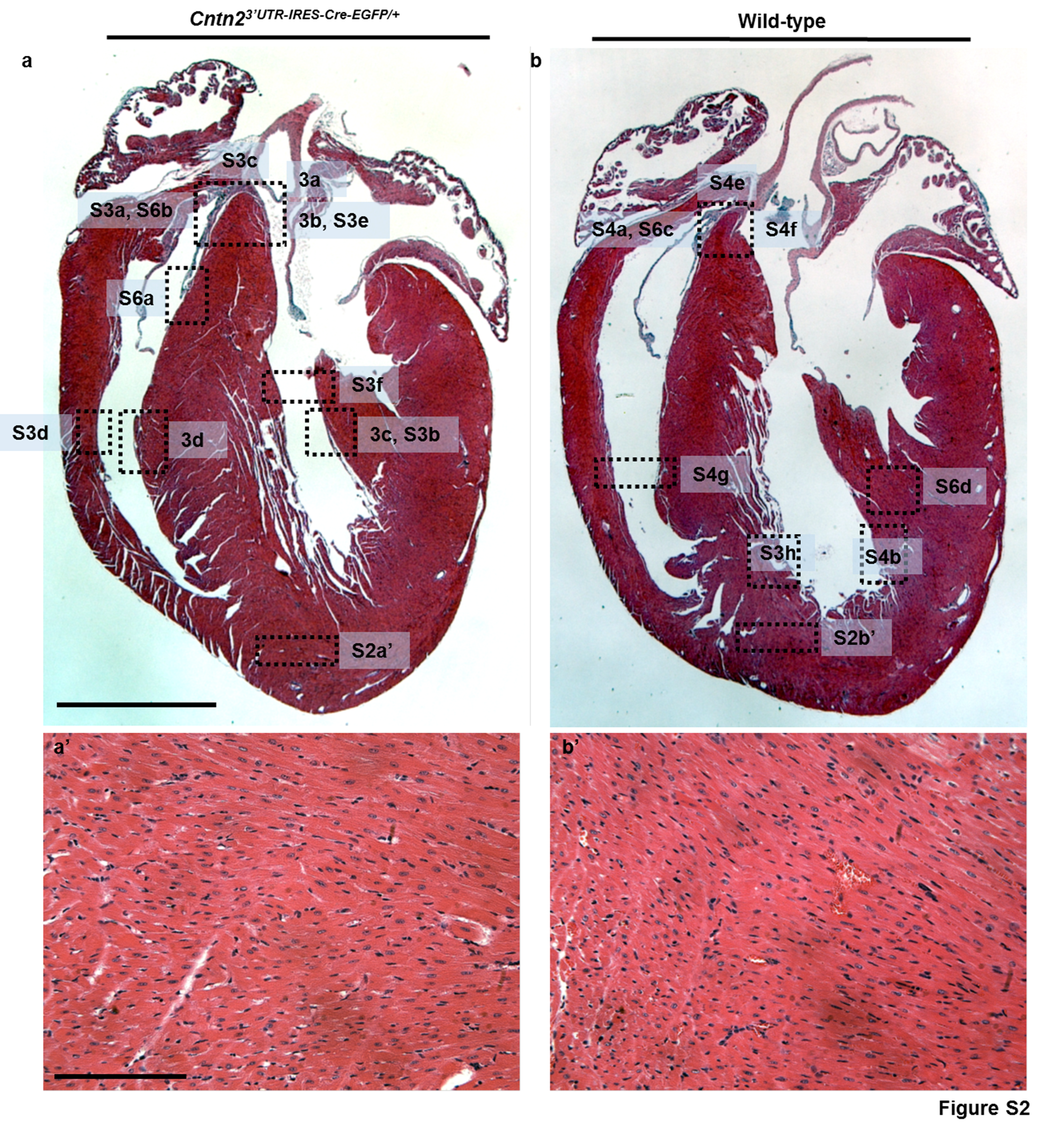

Supplement: S2 Fig — (a, b) Hematoxylin and Eosin (H&E) stained heart sections in a four-chamber orientation from (a) Cntn23’UTR-IRES-Cre-EGFP/+ and (b) WT animals at P42 showing the approximate anatomical location (1.25 X objective, black dotted inlets) of the detailed cardiac structures (dashed boxes) represented in Figs 3A–3D, S3, S4 and S6. (a’, b’) Higher magnification (20X objective, blue dotted inlets) images of H&E stained sections shown in (a, b) for confirming structural and morphological integrity of (a) Cntn23’UTR-IRES-Cre-EGFP/+ and (b) WT animals respectively. Scale bars: 500 μm. (TIF) [file pone.0174517.s002.tif]

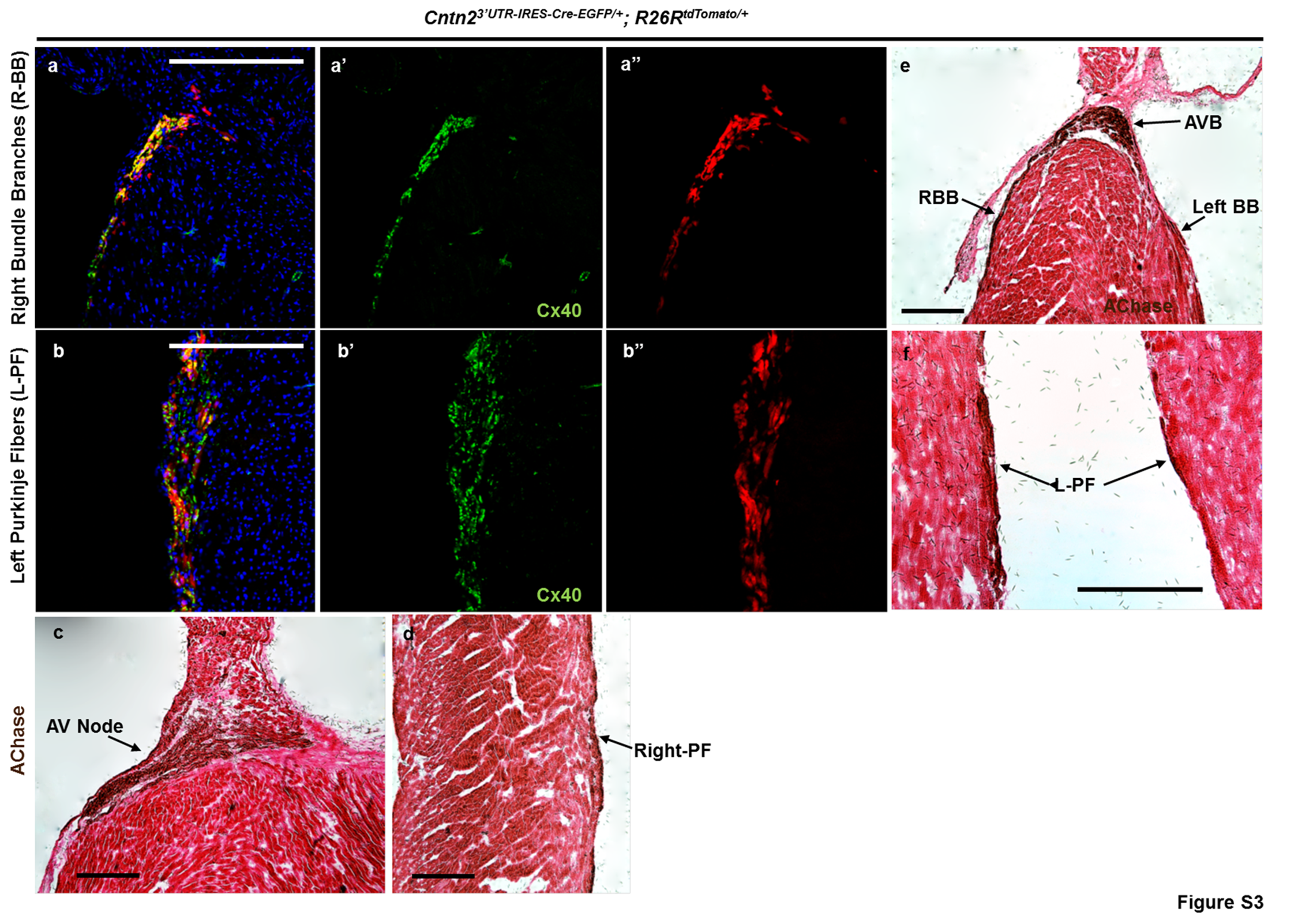

Supplement: S3 Fig — (a,b) High-power confocal images of consecutive heart sections of P42 Cntn23’UTR-IRES-Cre-EGFP/+; R26RtdTomato/+ mice to corroborate the co-expression (a and b) of the landmark Purkinje cell marker Cx40 (a’ and b’ in green) and native tdTomato (a” and b”‘ in red) in the right BB (a) and left PF network (b). Blue signal indicates nuclear counterstain by DAPI. (c-f) Acetylcholinesterase (AChase) staining of sister sections from a P42 Cntn23’UTR-IRES-Cre-EGFP/+; R26RtdTomato/+ heart, demonstrating clear identification of the AVN (c), right PFs (d), AVB and BBs (e), and left PFs (f). Scale bars: (a-b) 100 μm; (c-f) 500 μm. The precise anatomical location of the structures labeled in (a-f) are shown at lower magnification in S2A Fig. (TIF) [file pone.0174517.s003.tif]

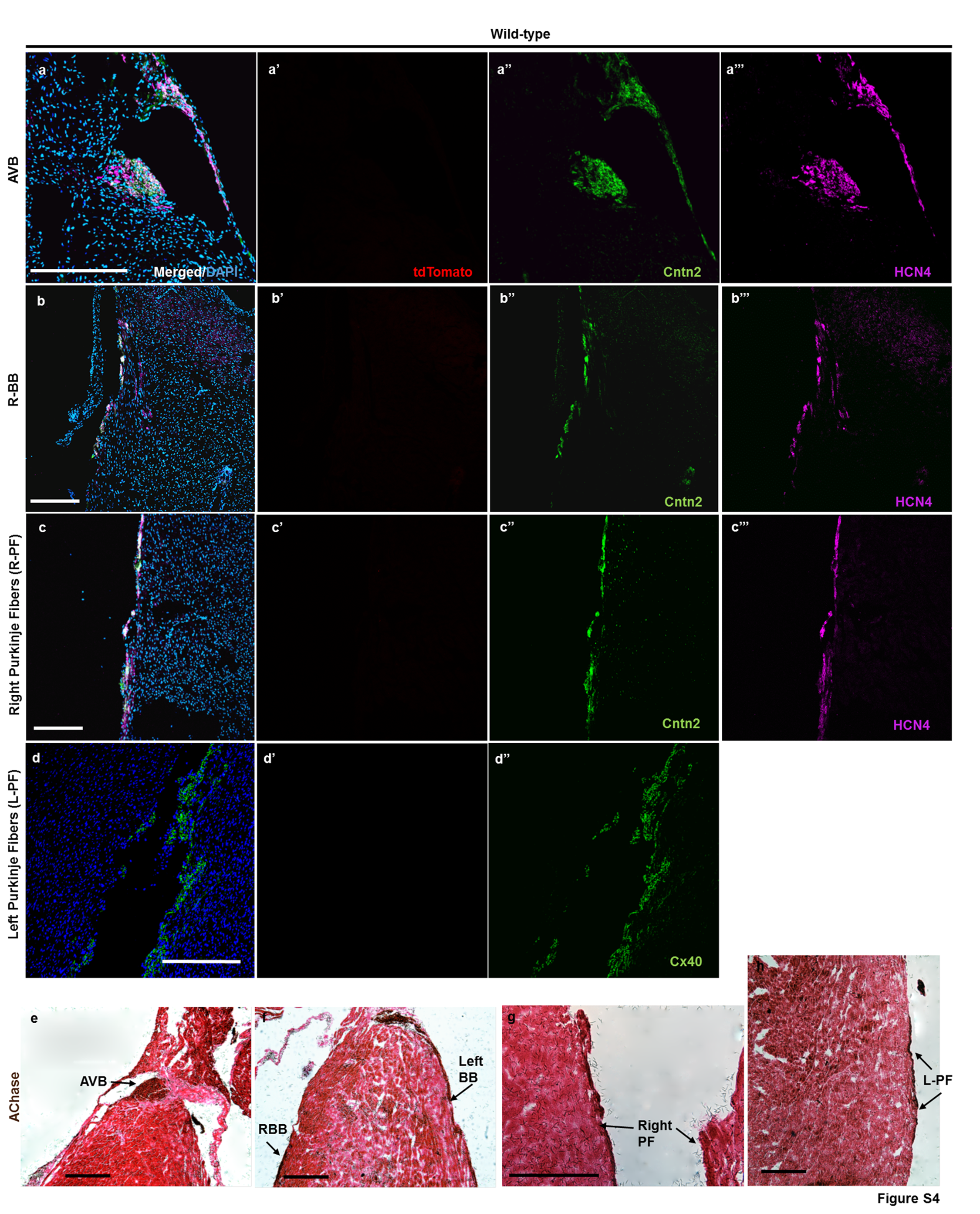

Supplement: S4 Fig — Littermates of Cntn23’UTR-IRES-Cre-EGFP/+; R26RtdTomato/+ mice at P42 were used as WT controls. (a, a’, a”, a”‘) (a) Merged image of (a’) tdTomato (red), (a”) Cntn2 (green), and (a”‘) Hcn4 (magenta) in the AV Bundle (AVB). (b, b’, b”, b”‘) (b) Merged image of (b’) tdTomato (red), (b”) Cntn2 (green), and (b”‘) Hcn4 (magenta) in the right Bundle Branch (R-BB). (c, c’, c”, c”‘) (c) Merged image of (c’) tdTomato (red), (c”) Cntn2 (green), and (c”‘) Hcn4 (magenta) in the right Purkinje Fibers (R-PF). (d, d’, d”) (d) Merged image of (d’) tdTomato (red) and (d”) Cx40 (green) in the left Purkinje Fibers (L-PF). As expected, we observed no evidence of tdTomato expression in the VCS (a’-d’), indicating a lack of recombination in WT animals. (e-h) AChase staining of sister sections clearly demarcating the (e) AVB, (f) left and right BBs, (g) RBB, and (h) left PFs. (a-d) Blue signal indicates nuclear counterstaining by DAPI. Scale bars: (a-d)100μm; (e-h) 500μm. The precise anatomical location of the structures labeled in (a-f) are shown at lower magnification in S2B Fig. (TIF) [file pone.0174517.s004.tif]

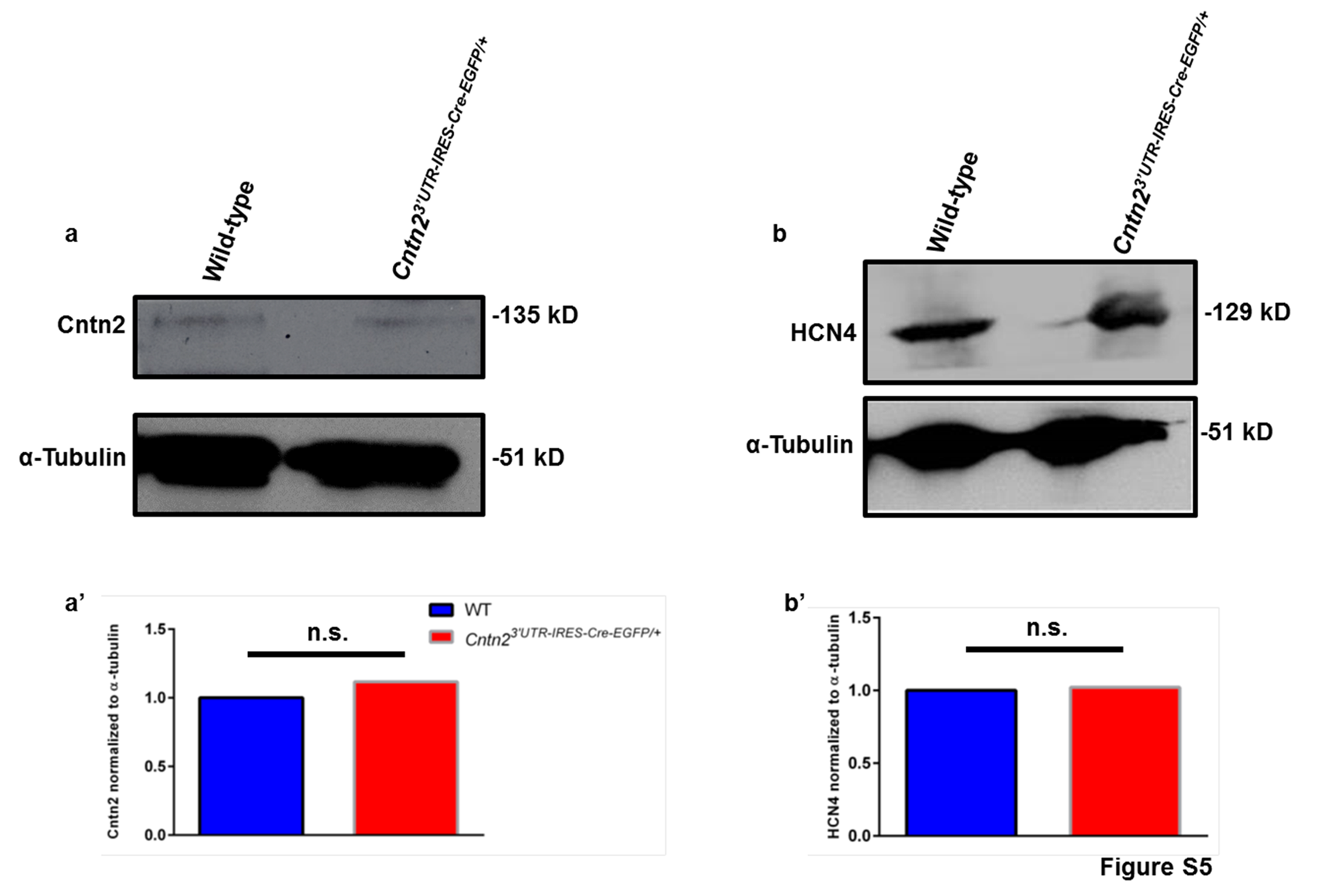

Supplement: S5 Fig — (a-b) Total protein was extracted from whole heart tissue of P42 Cntn23’UTR-IRES-Cre-EGFP/+ and WT mice (n = 3 hearts for each group), and the amount of total protein was normalized across samples with α-Tubulin serving as a loading control. a) Cntn2 protein expression was assessed by Western Blot analysis. a’) Quantification of the blots in (a) by ImageJ confirmed that Cntn2 expression is maintained in Cntn23’UTR-IRES-Cre-EGFP/+ mice. b) Hcn4 protein expression was evaluated by Western Blot analysis. b’) Quantification of the blots in (b) verified that Hcn4 expression is prserved in Cntn23’UTR-IRES-Cre-EGFP/+ mice. Quantification was based on two independent blots for each target protein. Blue and red bars represent protein quantification for WT and Cntn23’UTR-IRES-Cre-EGFP/+ mice, respectively. (TIF) [file pone.0174517.s005.tif]

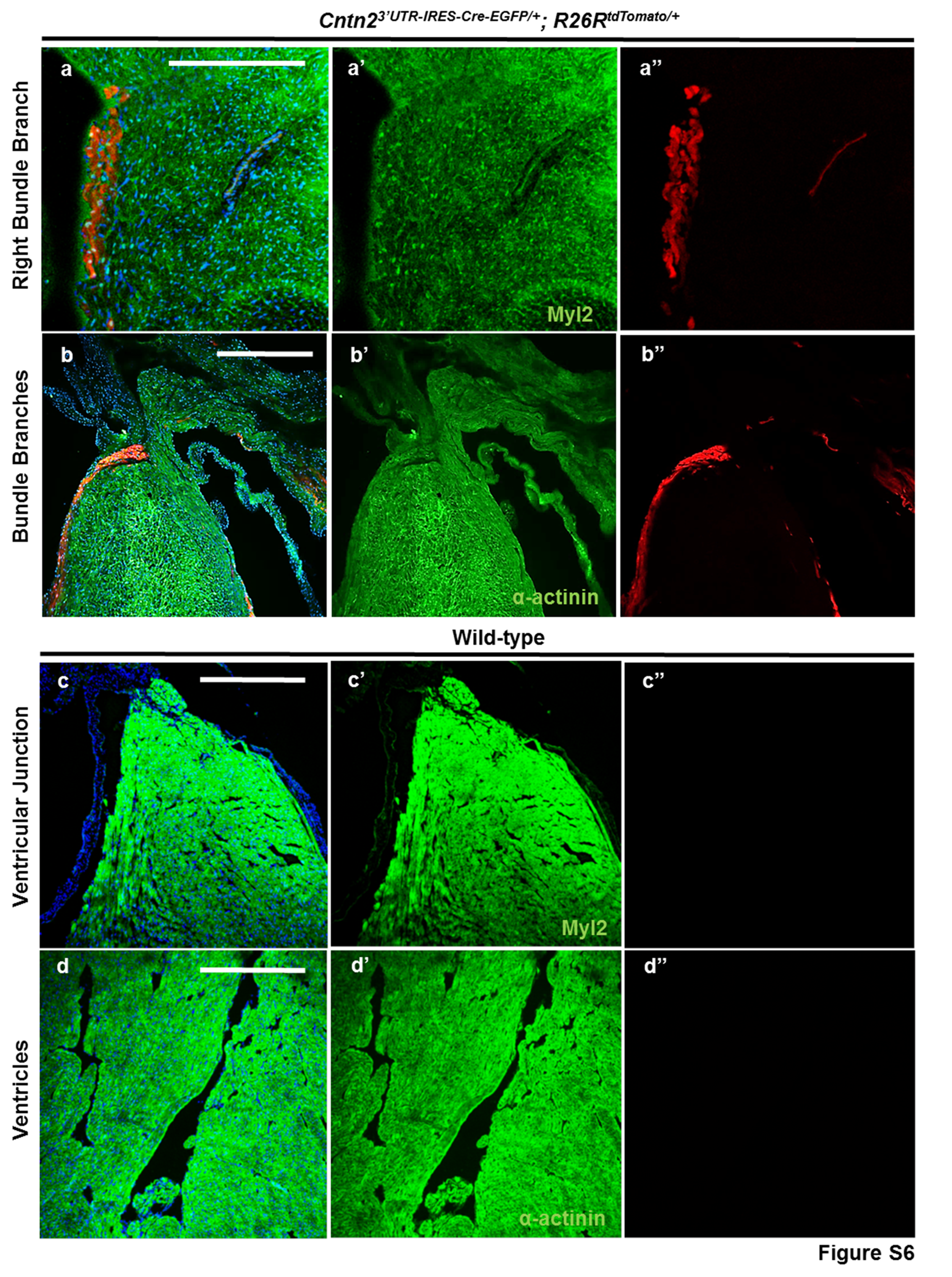

Supplement: S6 Fig — High-power confocal images of sections derived from Cntn23’UTR-IRES-Cre-EGFP/+; R26RtdTomato/+ (a-b) and WT (c-d) mice at P42. Sections were stained for either the pan-ventricular marker Myl2 (a’ and c’) or the pan-cardiac marker α-actinin (b’ and d’). No obvious differences in either marker were found between the two groups of mice. As expected, tdTomato expression was only observed in the presence of the Cntn23’UTR-IRES-Cre-EGFP/+ allele (compare a”-b” with c”-d”). Blue signal indicates nuclear counterstaining by DAPI. Scale bars: 100μm. The precise anatomical location of the structures labeled in (a-d) are shown at lower magnification in S2A and S2B Fig. (TIF) [file pone.0174517.s006.tif]

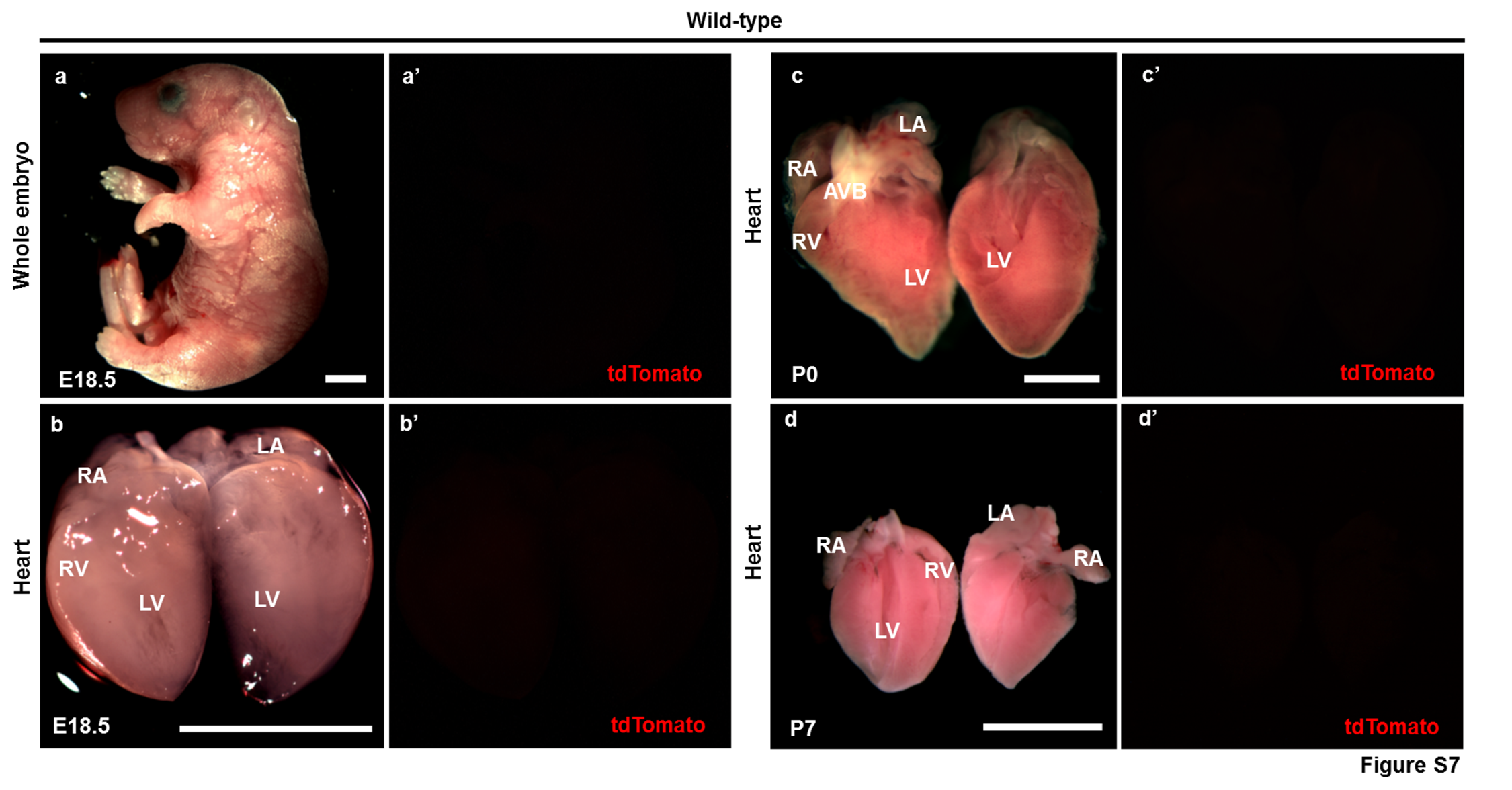

Supplement: S7 Fig — (a, a’) Whole mount fluorescent imaging of an E18.5 WT embryo exhibit no reporter expression indicating no recombination. We also confirmed no tdTomato expression in the brain of an E16.5 WT embryo (data not shown). (b, b’) After microdissection of a WT heart at E18.5, no reporter signal was observed. (c, c’) WT P0 mice hearts after dissection revealed no reporter protein expression. (d, d’) Microdissected P7 WT control hearts do not express tdTomato protein in any cardiac structures. n = 8 hearts were dissected for each developmental time point. Scale bar: 500 μm. (TIF) [file pone.0174517.s007.tif]

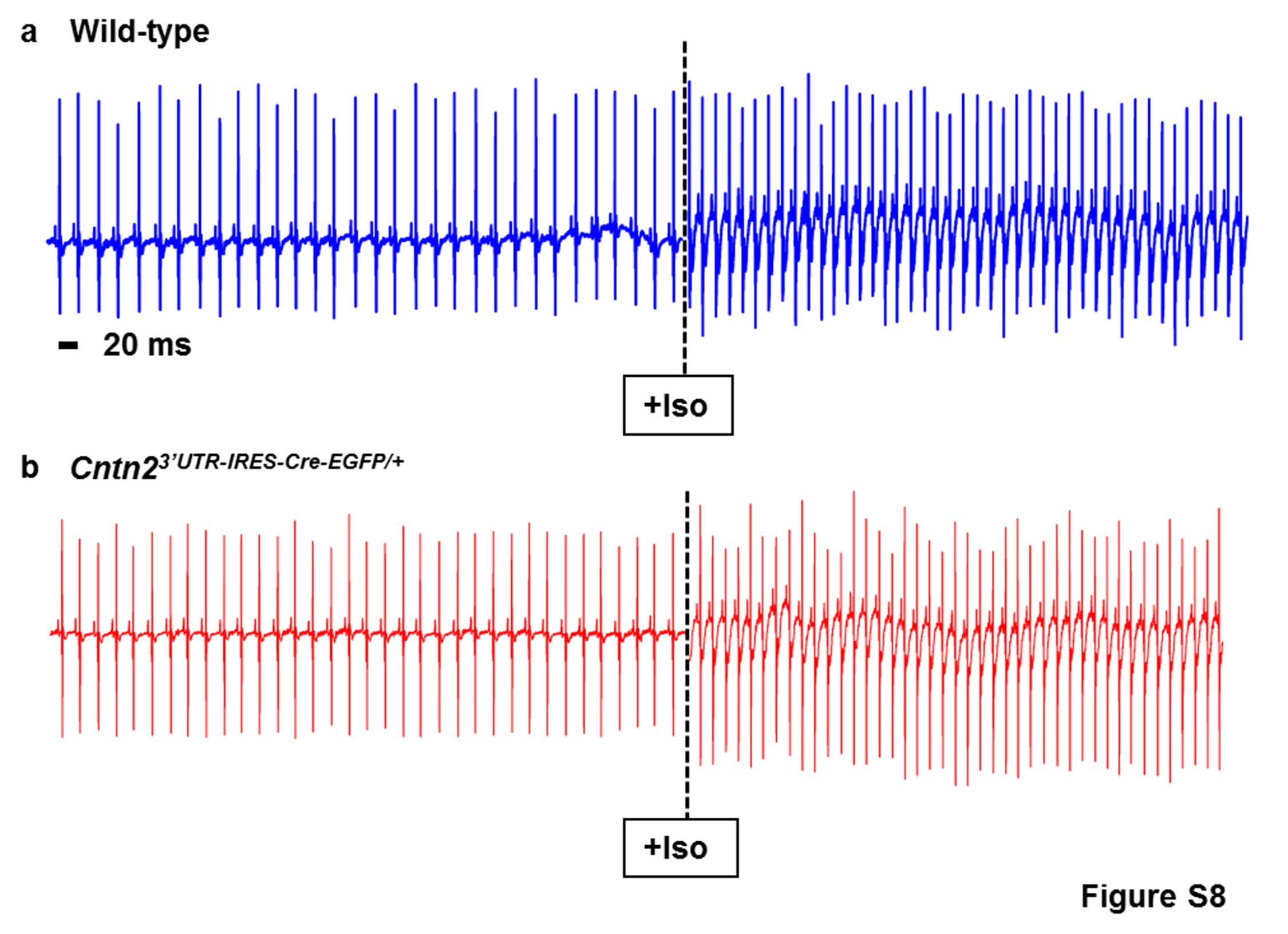

Supplement: S8 Fig — (a-b) Surface lead II ECGs were recorded continuously before and after pharmacological stress with isoproterenol. Representative ECG tracings are shown for (a) WT and (b) Cntn23’UTR-IRES-Cre-EGFP/+ mice. No non-sinus rhythms were observed in either group before or after isoproterenol injection (n = 9 per group). Scale bar: 20 milliseconds (ms). (TIF) [file pone.0174517.s008.tif]
