# Supplementary material for: Phenotypically silent Cre recombination within the postnatal ventricular conduction system
Source: PLoS One. 2017 Mar 30;12(3):e0174517. doi: 10.1371/journal.pone.0174517 (PMC5373586; doi:10.1371/journal.pone.0174517)
Supplement: S1 Table — (DOCX) [file pone.0174517.s009.docx]

**SUPPORTING INFORMATION**

**S1 Table. List of genotyping primers.**

| **Names of primers** | **Sequences of primers** |
| --- | --- |
| **F1** | **5' CTTCGCTTCTTAAGGCCTCCATATG 3'** |
| **R1** | **5' TTATTCCAAGCGGCTTCGGC 3'** |
| **F2** | **5' ATATTGCTGAAGAGCTTGGCGGC 3'** |
| **R2** | **5' TGTTAGGGTGGGTGTCCAGCTTG 3'** |
| **F3** | **5' GCTGCTGCCCGACAACCACTA 3'** |
| **R3** | **5' AGGGTACTGTTGGGAGAAGGTGTCT 3'** |
| **F4** | **5' GCATTACCGGTCGATGCAACGAGTGATGAG 3'** |
| **R4** | **5' GAGTGAACGAACCTGGTCGAAATCAGTGCG 3'** |
